# Hypoxia-induced MIR31HG expression promotes partial EMT and basal-like phenotype in pancreatic ductal adenocarcinoma based on data mining and experimental analyses

**DOI:** 10.1186/s12967-025-06292-x

**Published:** 2025-03-10

**Authors:** Ching-Chung Ko, Pei-Ming Yang

**Affiliations:** 1 Department of Medical Imaging, Chi Mei Medical Center, Tainan, 71004 Taiwan; 2 Department of Health and Nutrition, Chia Nan University of Pharmacy and Science, Tainan, 71710 Taiwan; 3 School of Medicine, College of Medicine, National Sun Yat-Sen University, Kaohsiung, 80424 Taiwan; 4 Graduate Institute of Cancer Biology and Drug Discovery, College of Medical Science and Technology, Taipei Medical University, No. 301, Yuantong Rd., Zhonghe Dist., New Taipei City, 235603 Taiwan; 5 PhD Program for Cancer Molecular Biology and Drug Discovery, College of Medical Science and Technology, Taipei Medical University and Academia Sinica, Taipei, 11031 Taiwan; 6 TMU Research Center of Cancer Translational Medicine, Taipei, 11031 Taiwan; 7 Cancer Center, Wan Fang Hospital, Taipei Medical University, Taipei, 11696 Taiwan; 8 Taipei Cancer Center, Taipei Medical University (TMU) and Affiliated Hospitals Pancreatic Cancer Groups, Taipei Medical University, Taipei, 11031 Taiwan

**Keywords:** Epithelial-mesenchymal transition, Hypoxia, Long non-coding RNA, MIR31HG, Pancreatic cancer

## Abstract

**Background:**

Pancreatic ductal adenocarcinoma (PDAC) is the most common and aggressive type of pancreatic cancer, with a five-year survival rate below 8%. Its high mortality is largely due to late diagnosis, metastatic potential, and resistance to therapy. Epithelial-mesenchymal transition (EMT) plays a key role in metastasis, enabling cancer cells to become mobile. Partial EMT, where cells maintain both epithelial and mesenchymal traits, is more frequent in tumors than complete EMT and contributes to cancer progression. The long non-coding RNA MIR31 host gene (*MIR31HG*) has recently emerged as a critical factor in PDAC oncogenesis. This study aimed to investigate *MIR31HG*’s role in partial EMT and its association with the basal-like PDAC subtype.

**Methods:**

We analyzed the relationship between *MIR31HG* expression, partial EMT, and the basal-like subtype of PDAC by integrating data from public databases. We reanalyzed public data from PDAC patient-derived organoids to assess *MIR31HG* expression and gene signatures under hypoxic and normoxic conditions. RNA sequencing and bioinformatics analyses, including gene set enrichment analysis (GSEA), were used to investigate differentially expressed genes and pathway enrichments. EMT, partial EMT, and hypoxia scores were calculated based on the expression levels of specific gene sets.

**Results:**

We observed that *MIR31HG* overexpression strongly correlates with higher partial EMT scores and the stabilization of the epithelial phenotype in PDAC. *MIR31HG* is highly expressed in the basal-like subtype of PDAC, which exhibits partial EMT traits. Hypoxia, a hallmark of basal-like PDAC, was shown to significantly induce *MIR31HG* expression, thereby promoting the basal-like phenotype and partial EMT. In patient-derived organoids, hypoxic conditions increased *MIR31HG* expression and enhanced basal-like and partial EMT gene signatures, while normoxia reduced these expressions. These findings suggest that hypoxia-induced *MIR31HG* expression plays a crucial role in driving the aggressive basal-like subtype of PDAC.

**Conclusions:**

Our results indicate that *MIR31HG* is crucial in regulating PDAC progression, particularly in the aggressive basal-like subtype associated with hypoxia and partial EMT. Targeting the *MIR31HG*-mediated network may offer a novel therapeutic approach to combat hypoxia-driven PDAC.

**Supplementary Information:**

The online version contains supplementary material available at 10.1186/s12967-025-06292-x.

## Background

The incidence of pancreatic cancer has significantly risen in recent years, making it the seventh leading cause of cancer-related mortality worldwide [1]. Due to its poor prognosis, the number of deaths among pancreatic cancer patients is nearly equivalent to the number of new cases [1]. Pancreatic ductal adenocarcinoma (PDAC) is the most common and aggressive form, comprising over 85% of cases [2], with a five-year survival rate of less than 8% [3]. Factors such as late-stage diagnosis, high metastatic potential, and drug resistance contribute to its high fatality rate [4]. Even patients diagnosed at an early stage face a substantial risk of recurrence [5]. Although surgical resection remains the only potential cure, 80–85% of cases are deemed unresectable. Additionally, nearly half of patients present with metastases at the time of diagnosis, precluding curative surgery [4]. For patients with advanced PDAC, treatment options like gemcitabine, nanoparticle albumin-bound paclitaxel, and FOLFIRINOX (a combination of fluorouracil, leucovorin, irinotecan, and oxaliplatin) offer limited clinical efficacy [6–10]. Consequently, there is an urgent need for the development of new therapeutic approaches.

Previous transcriptome-based research has identified several molecular subtypes of PDAC, each with distinct biological characteristics and impacts on patient survival outcomes [11–15]. Among these, the classical and basal-like subtypes have emerged as key classifications, shedding light on the disease’s heterogeneity and shaping personalized therapeutic approaches. The classical subtype is characterized by the expression of genes linked to epithelial differentiation and developmental signaling, such as GATA binding protein 6 (GATA6) and HNF1 homeobox A (HNF1A). It typically exhibits a more differentiated phenotype and shows better responsiveness to chemotherapy. Conversely, the basal-like subtype is defined by the activation of mesenchymal and stem-cell-like gene signatures, such as MYC proto-oncogene, bHLH transcription factor (MYC) and keratin 5 (KRT5), and is associated with an aggressive, undifferentiated phenotype. This subtype correlates with poorer outcomes and resistance to conventional therapies [11–15].

Epithelial-to-mesenchymal transition (EMT) is a biological process in which polarized epithelial cells transform into contractile and motile mesenchymal cells. This transition is marked by the loss of cell-cell adhesion and associated markers, such as E-cadherin, along with the increased expression of mesenchymal markers, including Vimentin and Fibronectin. Cells undergoing EMT also acquire motility and adopt fibroblast-like characteristics [16–20]. EMT plays a vital role during embryonic development by facilitating the formation of new tissues and organs. Additionally, it is implicated in the pathogenesis of tissue fibrosis, tumor progression, and metastasis [21–23]. More specifically, partial activation of the embryonic EMT program, known as partial EMT, is thought to be a key driver of tumor progression from initiation to metastasis and contributes to the development of drug resistance [24–26].

The lncRNA *MIR31HG*, formerly known as *LOC554202*, is the host gene for *miR-31*. Acting as an oncogene and a poor prognostic indicator, *MIR31HG* is overexpressed in various cancers, including PDAC [27–33]. *MIR31HG* targets several key molecules such as HIF-1α, p21, *miR-193*, and *miR-214*, contributing to tumor growth, metastasis, and chemotherapy resistance [27–30, 32]. As a co-activator of HIF-1α, it is also referred to as *LncHIFCAR* and has been shown to drive oral cancer progression [32]. Interestingly, *MIR31HG* is downregulated in some cancers, such as esophageal squamous cell carcinoma, hepatocellular carcinoma, and bladder cancer [34–36], indicating that its function may be cancer type-specific. To date, only one study has explored *MIR31HG*’s oncogenic role in PDAC [30]. Our previous bioinformatics study suggested that *MIR31HG* is overexpressed in PDAC and is associated with worse disease-free survival, promoting an oncogenic role by enhancing TGFβ-induced EMT [37]. Therefore, further research is needed to fully understand the oncogenic functions of *MIR31HG* in PDAC.

In this study, we investigated the role of *MIR31HG* in PDAC, particularly its association with partial EMT and the basal-like subtype. We found that *MIR31HG* overexpression correlates with higher partial EMT scores and helps stabilize the epithelial phenotype. Additionally, *MIR31HG* is highly expressed in the aggressive basal-like subtype of PDAC, which is characterized by partial EMT. Hypoxia, a common condition in basal-like PDAC, induces *MIR31HG* expression, further promoting the basal-like phenotype and partial EMT, suggesting that *MIR31HG* plays a pivotal role in PDAC progression under hypoxic conditions.

## Methods

### Cell culture

PANC-1 (#60284) cells from the Bioresource Collection and Research Center (BCRC; HsinChu, Taiwan) were kindly provided by Prof. Hsin-Yi Chen (Taipei Medical University). The cells were cultured in Dulbecco’s Modified Eagle Medium (DMEM; #11965084; Gibco; Grand Island, NY, USA) supplemented with 10% fetal bovine serum (FBS; #35-010-CV; Corning; Tewksbury, MA, USA), 2 mM L-glutamine (#25030081; Gibco), 1% non-essential amino acids (#11140050; Gibco), 1 mM sodium pyruvate (#11360070; Gibco), and 1% antibiotic-antimycotic solution (#15240062; Gibco). *MIR31HG*-overexpressing (*MIR31HG*-2X and *MIR31HG*-5X) PANC-1 cells were established and kindly provided by Prof. Tsui-Chin Huang (Taipei Medical University). The *MIR31HG*-2X and −5X represent cells with minimally and robustly overexpressed *MIR31HG* levels, respectively, compared to cells stably transfected with the vector control (PANC-1-Vector). These cells were maintained in a humidified 37 °C, 5% CO2 incubator.

### Real-time quantitative polymerase chain reaction (qPCR)

The total RNA was purified using the GENEzol TriRNA Pure Kit (#GZX100; Geneaid, New Taipei City, Taiwan). First-strand cDNA synthesis was carried out with the iScript cDNA Synthesis Kit (#1708891; Bio-Rad Laboratories, Hercules, CA, USA). PCR amplification was conducted with the IQ2 SYBR Green Fast qPCR System Master Mix (#DBU-006; Bio-Genesis Technologies, Taipei, Taiwan) on a QuantStudio1 Real-Time PCR System (Thermo Fisher Scientific, Waltham, MA, USA). The following primer pairs were used: *MIR31HG*, 5′-CACCAAGGTGTTCCTGCCTA-3′ and 5′-CAACCAGGCCAAAAGCATCC-3′; β-Actin, 5′-GTTGCTATCCAGGCTGTGCT-3′ and 5′-AGGGCATACCCCTCGTAGAT-3′. Primer specificity was confirmed by melt curve analysis. Relative expression levels relative to the reference gene (β-Actin) were calculated using the ΔΔCt method, with each reaction performed in triplicate to ensure technical reproducibility.

### RNA-sequencing (RNA-Seq) analysis

Total RNA from *MIR31HG*-overexpressing PANC-1 cells was sent to Biotools (New Taipei City, Taiwan) for RNA sequencing using the Illumina NovaSeq 6000 platform (San Diego, CA, USA), producing 150-bp paired-end reads. Differentially expressed genes (DEGs) were identified with DEGseq, applying a|fold change| > 2 and an adjusted p value < 0.05, with normalization performed using transcripts per million (TPM). An online tool Morpheus (https://clue.io/morpheus) was used to generate the heat map. The minimum and maximum expression values for each gene were used to map the values to colors.

### PDAC cohort

The gene expression profile and clinical data for 177 PDAC patients (TCGA-PAAD PanCancer Atlas dataset [38]) were obtained from The Cancer Genome Atlas (TCGA) via the cBioPortal website (https://www.cbioportal.org/) [39–41]. PDAC patients were classified into classical and basal-like subtypes using the PurIST classifier [42]. PurIST is an R package that provides a clinically robust, single-sample classifier for molecular subtyping of pancreatic cancer based on transcriptomic data [42]. GraphPad Prism 9 was used to plot the Kaplan-Meier survival curve. Differences between survival curves were evaluated using the log-rank test, with a 95% confidence interval.

### Gene set enrichment analysis (GSEA)

Gene set enrichment analysis (GSEA) was performed using the GSEA v4.3.3 software [43, 44]. For gene signatures correlated with *MIR31HG* expression, the “Pearson” metric was used to rank genes, while the “Signal2Noise” metric was applied for two-group comparisons. A total of 1,000 permutations were performed, and gene sets with an FDR < 0.05 were considered significantly enriched. Other default parameters were applied unless otherwise specified. The following gene signatures were used: hallmarks for EMT and hypoxia [45], epithelial (KS_Epithelial) and mesenchymal (KS_Mesenchymal) genes [46], partial EMT genes (Partial_EMT) [47], and Moffitt’s classical (Classical_Sig) and basal-like (Basal-like_Sig) genes [12]. An online tool Venny 2.1 (https://bioinfogp.cnb.csic.es/tools/venny/) was used to generate the Venn diagram. Input gene sets were derived from significantly enriched GSEA results.

### Analyses for EMT, partial EMT, and hypoxia scores

The EMT score is calculated by adding the expression levels of mesenchymal genes (*CDH2*,* FN1*, *SNAI1*, *SNAI2*, *TWIST1*, *TWIST2*, *VIM*, *ZEB1*, and *ZEB2*) and subtracting the sum of the expression levels of epithelial genes (*CDH1*, *CLDN4*, *CLDN7*, *TJP3*, *MUC1*, and) [48]. Gene expression levels were log2-transformed prior to score calculation, and missing data were excluded from the analysis. The partial EMT score is determined by summing the expression levels of genes associated with partial EMT (*LAMB3*, *LAMC2*, and *PDPN*) [49]. Hypoxia scores (Buffa [50], Winter [51], and Ragnum [52]) for PDAC patients (TCGA-PAAD dataset) were retrieved from the cBioPortal website [39–41]. Scores were used without additional preprocessing and analyzed independently.

## Results

### MIR31HG overexpression highly correlates with partial EMT in PDAC

**Fig. 1:**
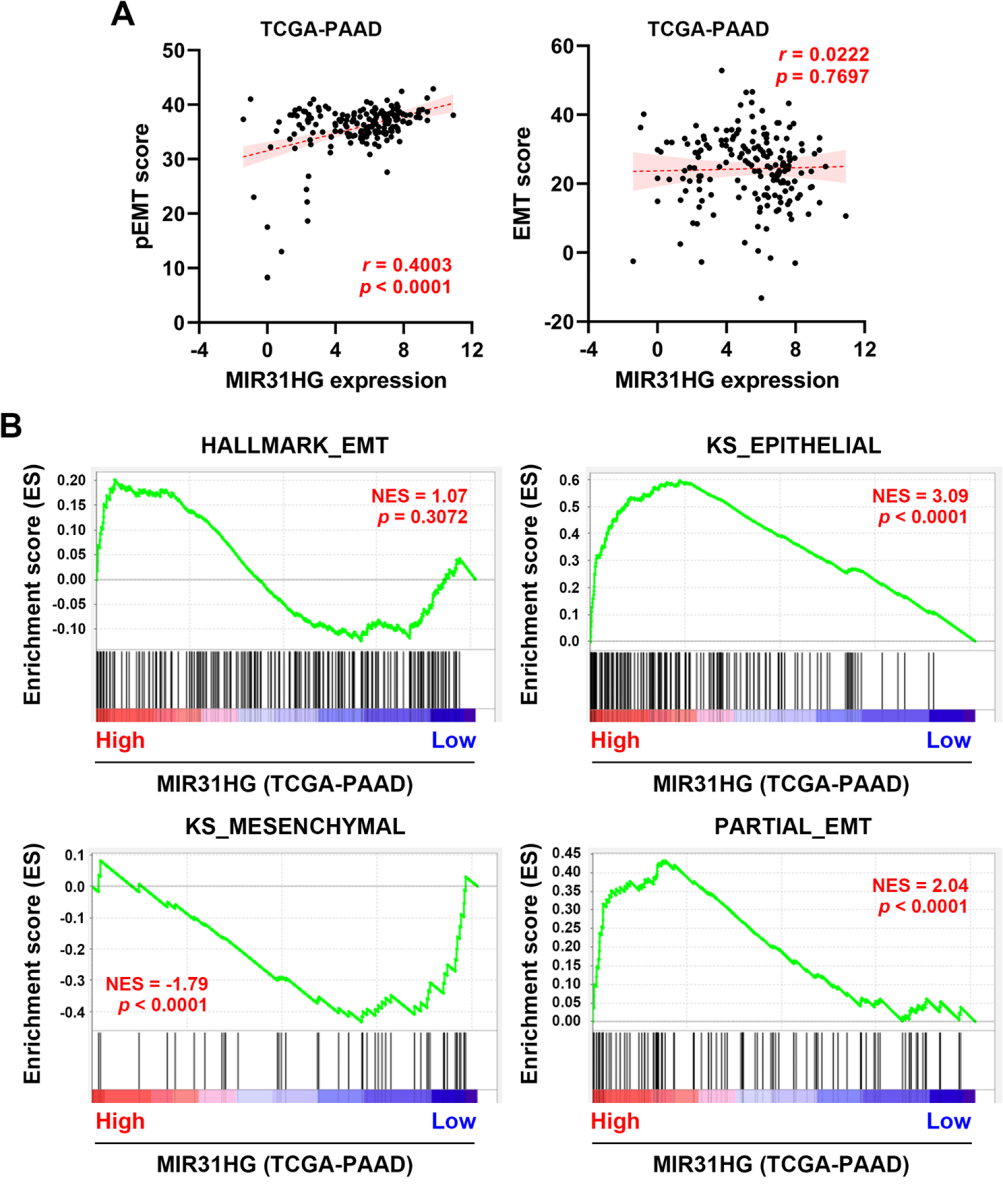
The correlation between MIR31HG and partial EMT in PDAC patients. (**A**) Data from PDAC patients (TCGA-PAAD dataset; *n* = 177) were obtained from the cBioPortal website (https://www.cbioportal.org/). EMT and partial EMT (pEMT) scores were calculated as described in the Materials and Methods section, and their relationship to MIR31HG gene expression was analyzed using Pearson’s correlation. **(B)** GSEA was performed to compare the gene sets (from left to right: Hallmark_EMT, KS_Epithelial, KS_Mesenchymal, and Partial_EMT) with MIR31HG expression in PDAC patients. NES, normalized enrichment score

Although we have demonstrated a promoting role of *MIR31HG* in TGFβ-induced complete EMT in PDAC cells, transient overexpression or knockdown of *MIR31HG* alone is not sufficient to alter the expression of common EMT markers, such as E-cadherin (*CDH1*) and Vimentin (*VIM*) [37]. We speculated that *MIR31HG* may contribute to the hybrid EMT state (partial EMT). To support this idea, the transcriptome data of PDAC patients (TCGA-PAAD dataset) were used to calculate the EMT and partial EMT scores. As shown in Fig. 1A, *MIR31HG*-high PDAC patients have a higher partial EMT score, and no significant correlation between *MIR31HG* and the EMT score was found. A similar relationship was also observed when comparing across different age groups ( ≦ 65 and > 65) (Supplementary Fig. S1). Additionally, gene set enrichment analysis (GSEA) was performed to measure the expression of EMT hallmark, epithelial/mesenchymal genes, and partial EMT gene signatures in *MIR31HG*-high and *MIR31HG*-low PDAC patients. Interestingly, epithelial and partial EMT gene signatures were enriched in *MIR31HG*-high PDAC patients (Fig. 1B), indicating that *MIR31HG* overexpression may be responsible for the stabilization of the epithelial phenotype during partial EMT and subsequent metastasis, as reported previously [53].

**Fig. 2:**
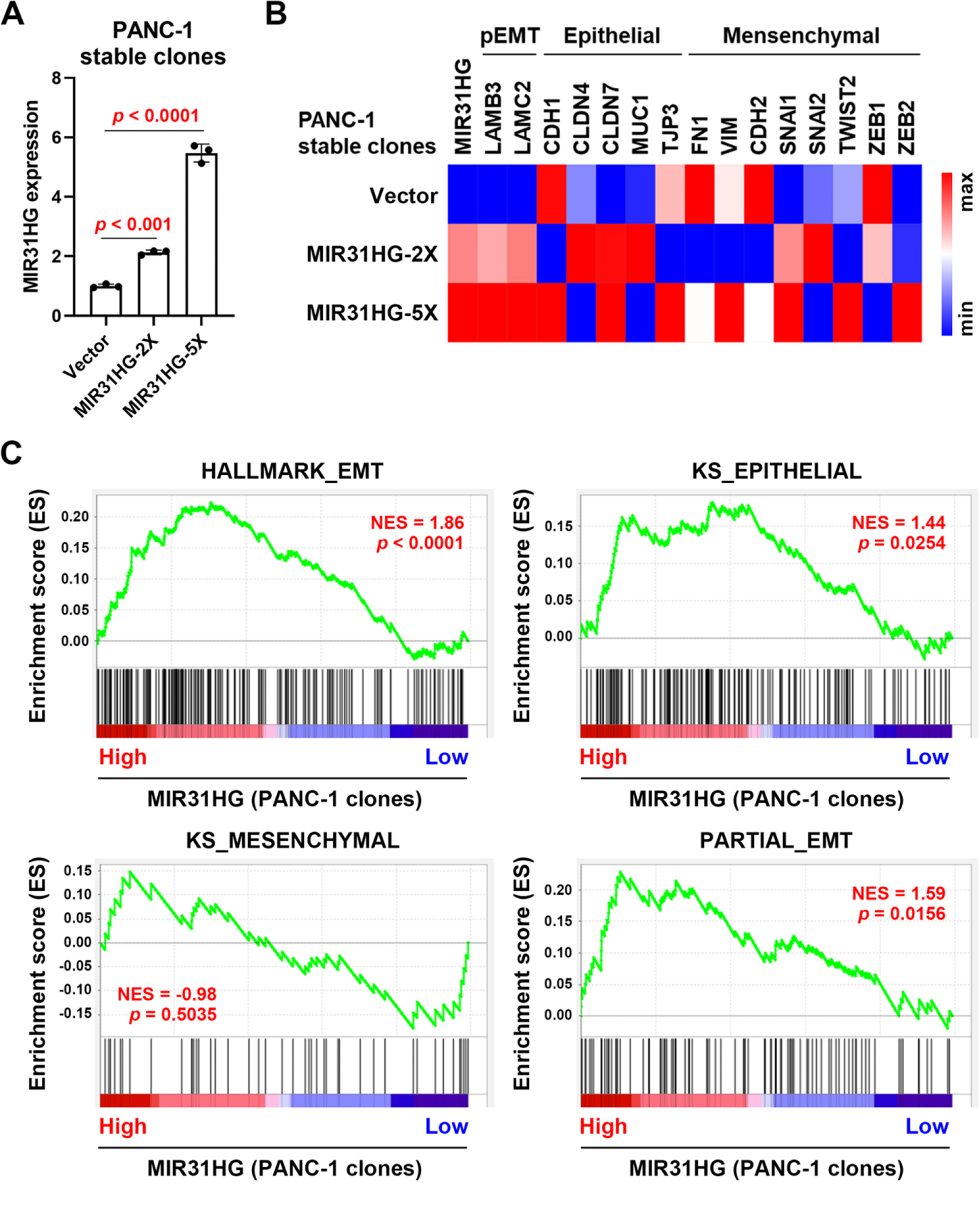
The correlation between MIR31HG and partial EMT in PANC-1 stable clones. (**A**) PANC-1 stable clones with minimal and robust MIR31HG overexpression (MIR31HG-2X and − 5X, respectively) were established. The relative MIR31HG expression was quantified by real-time qPCR. **(B)** Total RNA from PANC-1-Vector, PANC-1-MIR31HG-2X, and PANC-1-MIR31HG-5X cells was subjected to RNA-Seq analysis. The heat map shows the relative gene expressions for partial EMT (pEMT), epithelial, and mesenchymal markers. The minimum (blue) and maximum (red) expression values for each gene were used to map the values to colors. **(C)** GSEA was performed to compare the gene sets (from left to right: Hallmark_EMT, KS_Epithelial, KS_Mesenchymal, and Partial_EMT) with MIR31HG expression in PANC-1 stable clones. NES, normalized enrichment score

To confirm the above analysis, PANC-1 stable clones with different overexpressing levels of *MIR31HG* (*MIR31HG*-2X and − 5X), compared to the vector control (PANC-1-Vector), were established (Fig. 2A) and subjected to RNA sequencing (RNA-Seq) analysis. *MIR31HG* overexpression increased the levels of two partial EMT markers (*LAMB3* and *LAMC2*), especially in *MIR31HG*-5X PANC-1 cells (Fig. 2B). Consistent with the results in PDAC patients (Fig. 1B), epithelial and partial EMT gene signatures were enriched in *MIR31HG*-overexpressing PANC-1 cells (Fig. 2C). Furthermore, elevated LAMB3 and LAMC2 protein levels were confirmed in *MIR31HG*-2X and − 5X cells, without affecting E-cadherin and vimentin protein expression (**Supplementary Fig. S2**). Therefore, these results suggest that *MIR31HG* overexpression may promote partial EMT.

### The basal-like subtype of PDAC is characterized by partial EMT

**Fig. 3:**
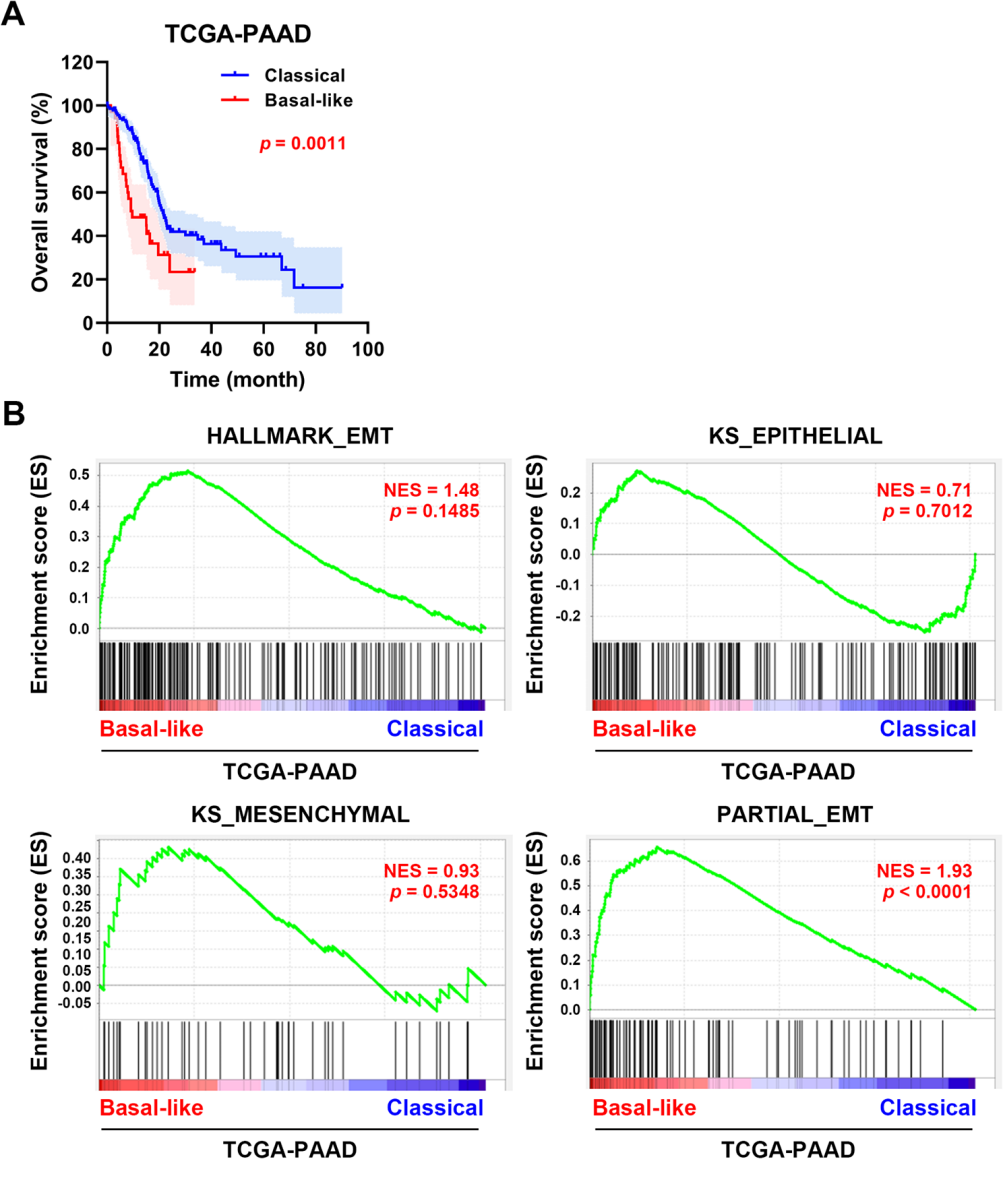
The correlation between partial EMT and PDAC subtypes. (**A**) Data from PDAC patients (TCGA-PAAD dataset; *n* = 177) were obtained from the cBioPortal website (https://www.cbioportal.org/). Patients were classified into classical (*n* = 142) and basal-like (*n* = 35) subtypes to compare their overall survivals. **(B)** GSEA was performed to compare the gene sets (from left to right: Hallmark_EMT, KS_Epithelial, KS_Mesenchymal, and Partial_EMT) in PDAC subtypes. NES, normalized enrichment score

It has been found that the basal-like subtype of PDAC is characterized by higher EMT gene expression [14]. To confirm this feature, PDAC patients (TCGA-PAAD dataset) were classified into classical and basal-like subtypes. Among 177 PDAC patients, 80% and 20% belonged to the classical and basal-like subtypes, respectively. No differences were observed between the two subtypes regarding age, gender, T/N/M factors, and stages (Table 1), suggesting that the distribution of cases was similar after subtyping. Consistent with a previous finding [12], basal-like subtype patients displayed worse overall survival compared to classical subtype patients (Fig. 3A). Although there was a trend for higher expression of EMT hallmark and mesenchymal genes in the basal-like subtype, the enrichment results were not statistically significant, and only the partial EMT gene signature was selectively enriched in the basal-like subtype (Fig. 3B). Therefore, partial EMT may contribute to the aggressive behavior of basal-like PDAC patients.

**Table 1:** Association of PDAC subtypes and clinical parameters

Parameters	Classical (*n* = 142)	Basal-like (*n* = 35)	*p* value
Age (years) ≦65 >65	75 67	19 16	0.8769
Gender Female Male	64 78	16 19	0.9436
T factor 1 + 2 3 + 4	25 117	7 28	0.7413
N factor 0 1 + 2	42 100	12 23	0.5877
M factor 0 1	138 4	35 0	0.3151
Stage Early (I + II) Late (III + IV)	136 6	33 2	0.7043

### Higher MIR31HG expression in basal-like PDAC subtype

**Fig. 4:**
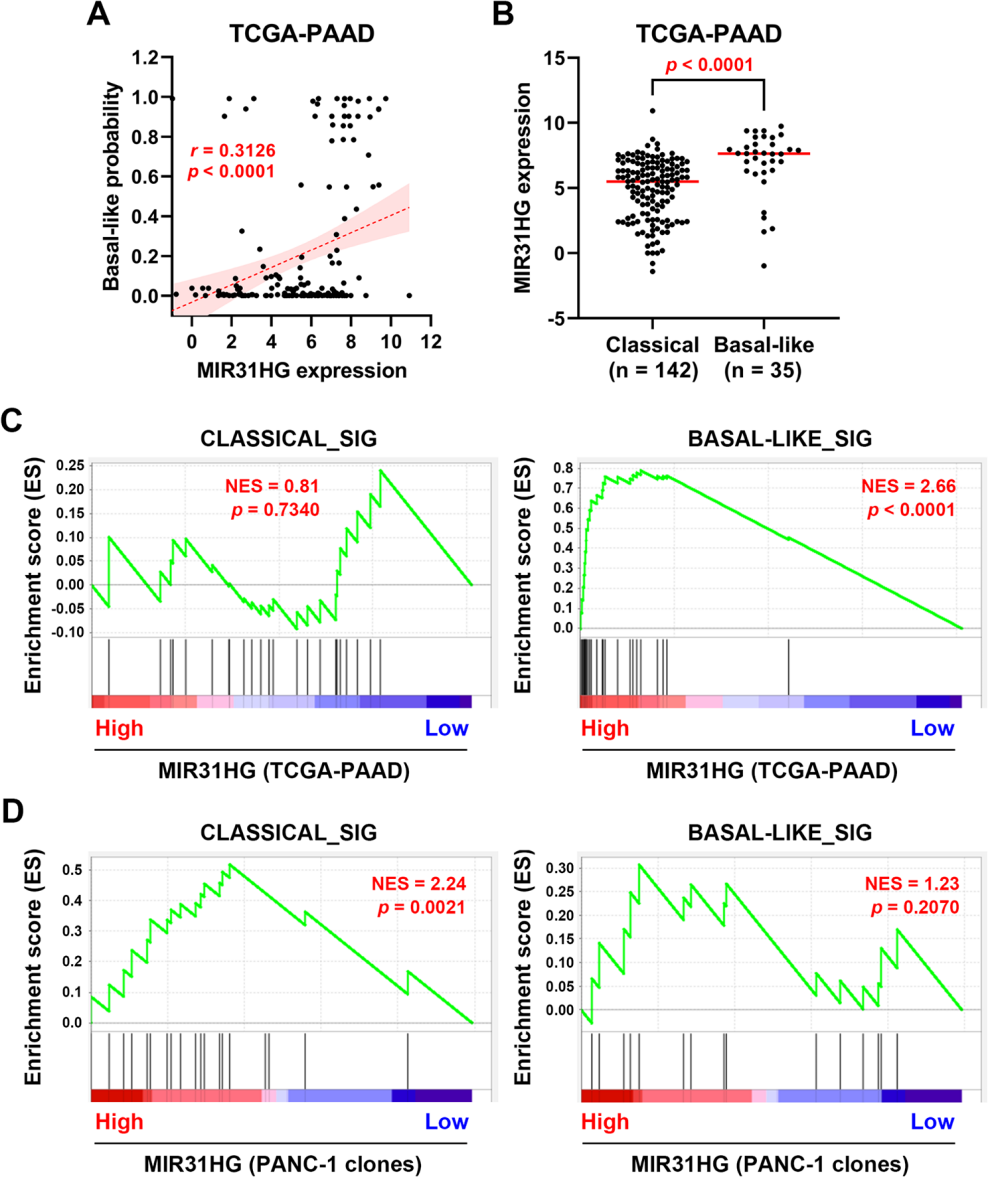
The correlation between MIR31HG and PDAC subtypes. (**A**) Data from PDAC patients (TCGA-PAAD dataset; *n* = 177) were obtained from the cBioPortal website (https://www.cbioportal.org/). The basal-like probability was calculated using the PurIST classifier and plotted against MIR31HG expression. Their relationship was analyzed using Pearson’s correlation. **(B)** PDAC patients were classified into classical (*n* = 142) and basal-like (*n* = 35) subtypes to compare their MIR31HG expressions. **(C**, **D)** GSEA was performed to compare the PDAC subtype gene signatures (left: classical; right: basal-like) with MIR31HG expression in PDAC patients (**C**) and in PANC-1 stable clones (**D**). NES, normalized enrichment score

The above analyses prompted us to further investigate whether *MIR31HG* overexpression can induce the basal-like subtype in PDAC. We found that *MIR31HG* expression was positively correlated with the basal-like probability in PDAC patients, as estimated by either a linear regression model (Fig. 4A) or a non-linear regression model (**Supplementary Fig. S3**). Consistently, *MIR31HG* expression was higher in basal-like PDAC patients (Fig. 4B). In addition, the basal-like gene signature was significantly enriched in *MIR31HG*-high PDAC patients (Fig. 4C). To determine whether *MIR31HG* overexpression drives a basal-like phenotype in PDAC, we analyzed data from *MIR31HG*-overexpressing PANC-1 cells. However, *MIR31HG* overexpression in PANC-1 cells was not sufficient to induce the expression of the basal-like gene signature (Fig. 4D, right panel), suggesting that *MIR31HG* alone does not induce the basal-like subtype in PDAC. Contrarily, the classical gene signature was enriched in *MIR31HG*-overexpressing PANC-1 cells (Fig. 4D, left panel). Because the classical PDAC subtype is characterized by high expression of epithelial and adhesion-associated genes [11], the enrichment results here were consistent with the finding that *MIR31HG* overexpression may stabilize the epithelial phenotype during partial EMT (Figs. 1B and 2C).

### Hypoxia induces MIR31HG expression and promotes the basal-like subtype in PDAC

**Fig. 5:**
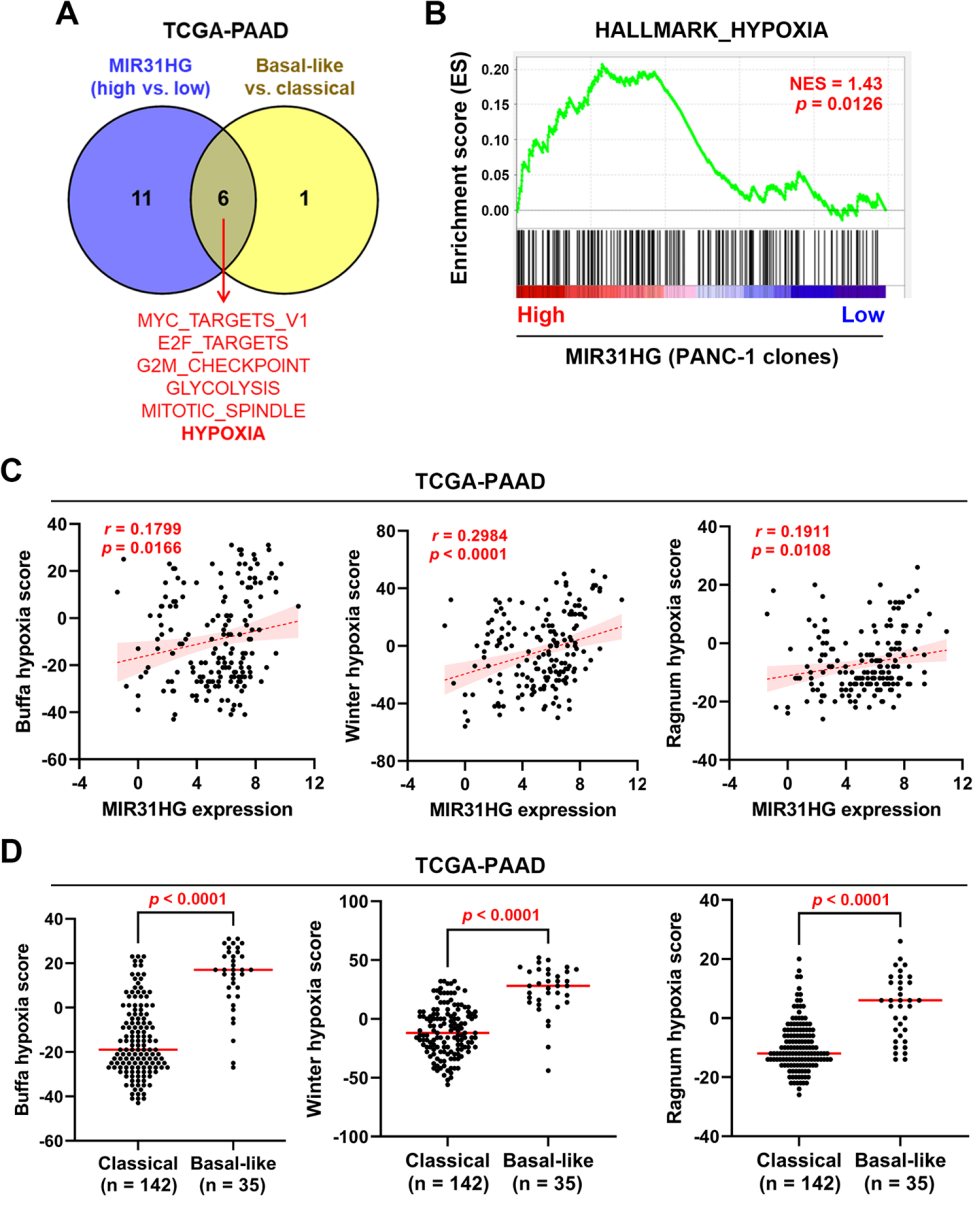
The association of hypoxia with MIR31HG and PDAC subtypes. (**A**) The overlapping cancer hallmarks enriched in MIR31HG-high vs. MIR31HG-low group and basal-like vs. classical group in PDAC patients. **(B)** The enrichment plot for relationship between the hypoxia cancer hallmark and MIR31HG expression in PANC-1 stable clones. NES, normalized enrichment score. **(C**,**D)** Data from PDAC patients (TCGA-PAAD dataset; *n* = 177) were obtained from the cBioPortal website (https://www.cbioportal.org/). The correlation between hypoxia scores and MIR31HG expression was plotted in C, and their relationship was calculated using Pearson’s correlation. The hypoxia scores in classical and basal-like subtypes were plotted in D

To explore the potential mechanism linking *MIR31HG* to PDAC subtypes, we compared the cancer hallmark enrichment results between *MIR31HG*-high vs. *MIR31HG*-low and basal-like subtype vs. classical subtype in the TCGA-PAAD dataset. As shown in Fig. 5A, the cancer hallmarks commonly enriched in both groups were MYC_TARGETS_V1, E2F_TARGETS, G2M_CHECKPOINT, GLYCOLYSIS, MITOTIC_ SPINDLE, and HYPOXIA. Because *MIR31HG* is identified as a HIF-1α co-activator [32], we further investigated the role of hypoxia. We confirmed that *MIR31HG* overexpression induced the expression of hypoxia-related genes in PANC-1 cells (Fig. 5B), consistent with the previous observation that *MIR31HG* overexpression induces a pseudo-hypoxia gene signature [32]. Supportively, *MIR31HG* expression was correlated with hypoxia scores (Buffa [50], Winter [51], and Ragnum [52]) in PDAC patients (Fig. 5C). The basal-like/squamous subtype is characterized by hypoxia [13]. We also showed that basal-like PDAC patients have higher hypoxia scores (Fig. 5D). Therefore, we hypothesized that hypoxia-induced *MIR31HG* expression may contribute to the aggressive behavior of the basal-like PDAC subtype.

**Fig. 6:**
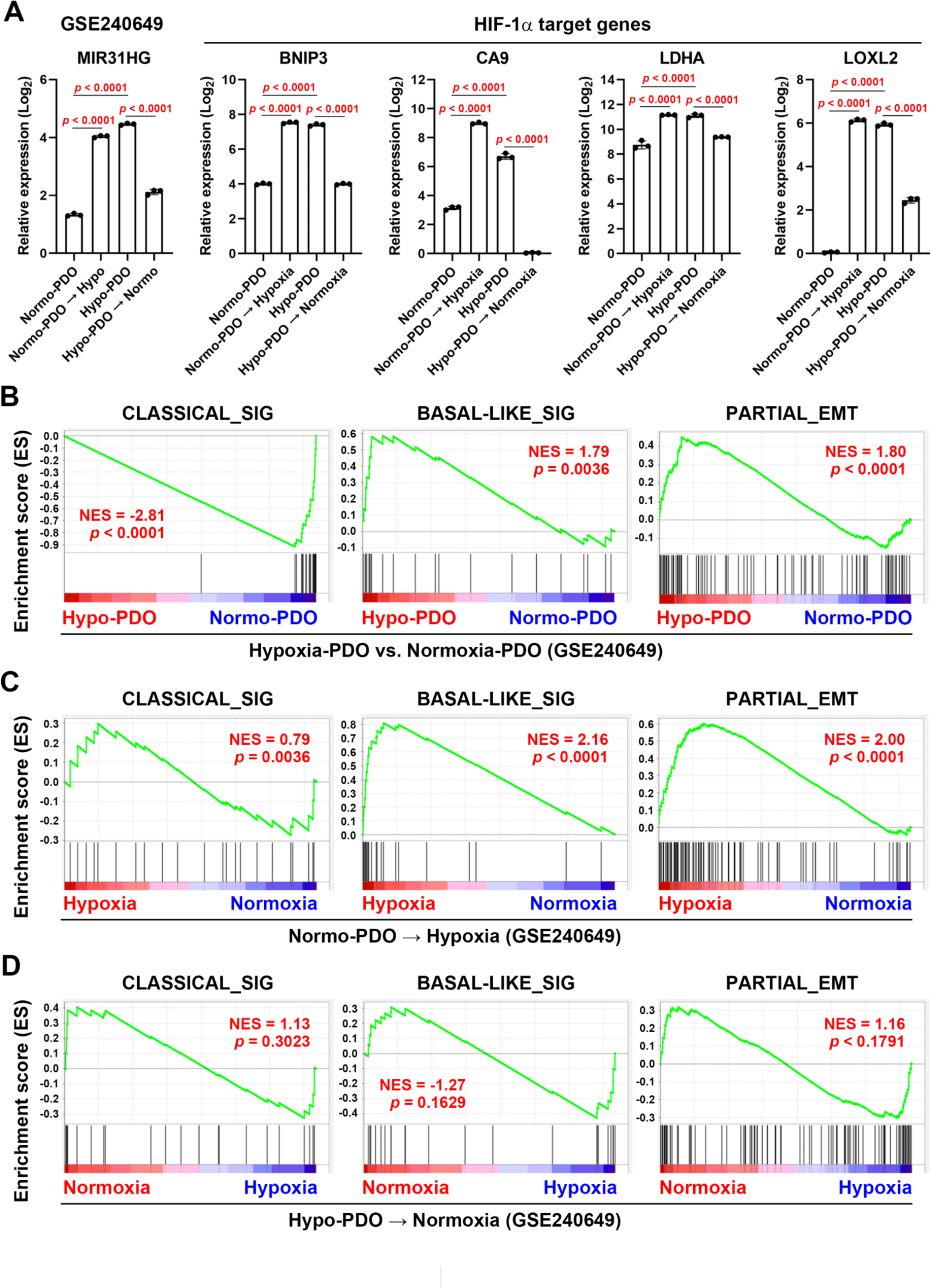
The role of hypoxia in MIR31HG expression, partial EMT and PDAC subtypes. (**A**) PDAC patient-derived organoid data were obtained from the NCBI GEO database (GSE240649). Organoids were established under normoxia (Normo-PDO) and hypoxia (Hypo-PDO) conditions, then switched to hypoxia and normoxia, respectively. The expression levels of MIR31HG and selected HIF-1α-target genes in each condition were plotted. **(B**-**D)** GSEA was performed to compare the enrichment of classical (left), basal-like (middle), and partial EMT (right) gene signatures in the Hypo-PDO vs. Normo-PDO group (**B**), hypoxia vs. normoxia in Normo-PDO (**C**), and normoxia vs. hypoxia in Hypo-PDO (**D**). NES, normalized enrichment score

To support the above analyses, we obtained the gene expression profiles of PDAC patient-derived organoids established under normoxia (20% O_2_) and hypoxia (1% O_2_) conditions (GSE240649 [54]). The effect of hypoxia was confirmed by the changes of several HIF-1α-target genes [32], including *BCL2 interacting protein 3* (*BNIP3*), *carbonic anhydrase 9* (*CA9*), *lactate dehydrogenase A* (*LDHA*), and *lysyl oxidase like 2* (*LOXL2*) (Fig. 6A) We found that organoids established under hypoxia (Hypo-PDO) have higher levels of *MIR31HG* and HIF-1α-target genes compared to those established under normoxia (Normo-PDO) (Fig. 6A). In addition, Hypo-PDO expressed higher basal-like and partial EMT gene signatures (Fig. 6B). Changing Normo-PDO to hypoxia resulted in *MIR31HG* and HIF-1α-target gene upregulation and the enrichment of basal-like and partial EMT gene signatures (Fig. 6A, C). In contrast, changing Hypo-PDO to normoxia reduced the expressions of *MIR31HG* HIF-1α-target gene (Fig. 6A), as well as basal-like/partial EMT gene signatures (Fig. 6D). Taken together, we concluded that hypoxia promotes the partial EMT and basal-like subtype in PDAC, with *MIR31HG* upregulation potentially playing an essential role in maintaining the epithelial phenotype during partial EMT.

## Discussion

Our findings highlight the pivotal role of *MIR31HG* in regulating partial EMT, a state in which cancer cells exhibit both epithelial and mesenchymal traits, facilitating metastasis and therapy resistance. The enrichment of partial EMT gene signatures in *MIR31HG*-overexpressing cells suggests that this lncRNA contributes to cellular plasticity, enabling tumor cells to adapt to microenvironmental stressors such as hypoxia. Unlike complete EMT, where cells lose their epithelial identity, partial EMT allows cancer cells to retain adhesion properties, aiding in both tumor dissemination and colonization of distant tissues. This dual functionality of *MIR31HG* supports the notion that partial EMT is not just a transitional state but a stable phenotype that drives PDAC aggressiveness.

The extracellular matrix (ECM) is a non-cellular structure within tissues that provides critical biochemical and mechanical support to surrounding cells. It consists of key glycoproteins, such as collagens, laminins, and fibronectins, which interact with various cell surface receptors like integrins and cadherins. These interactions regulate vital cellular processes, including communication, proliferation, adhesion, and migration [55]. Recent research suggests that increased ECM stiffness can trigger mechanotransduction pathways, promoting EMT in carcinoma cells [55]. Our previous study highlights the role of *MIR31HG* in facilitating EMT induced by TGFβ. However, altering *MIR31HG* levels (via knockdown or overexpression) did not consistently affect typical EMT markers, such as E-cadherin and vimentin, except for collagen type I alpha 1 chain (COL1A1) [37]. Whether COL1A1 plays a major role in *MIR31HG*-mediated partial EMT and basal-like phenotype in PDAC warrants further investigations.

A previous study revealed that *MIR31HG* plays a crucial role in regulating HIF-1 target genes [32]. Mechanistically, *MIR31HG* directly binds to the HIF-1α subunit, forming a complex that stabilizes HIF-1α at gene promoters. Acting as a molecular scaffold, *MIR31HG* facilitates the recruitment of both HIF-1α and the transcriptional coactivator p300 to hypoxia-responsive gene promoters. p300, known for its histone acetyltransferase activity, remodels chromatin into an open conformation conducive to transcriptional activation [32]. By regulating HIF-1 target gene expression, *MIR31HG* may promote hypoxia-induced partial EMT and the basal-like subtype in PDAC.

While the current analysis leverages data from TCGA, GEO, and cell line models, which provide valuable initial insights, their inherent limitations in sample size and representativeness restrict the generalizability of the results. Expanding the study to include real-world patient samples could address these limitations by providing a more comprehensive and clinically relevant dataset. Real-world samples would enable the assessment of findings across diverse populations and capturing the heterogeneity of clinical presentations and environmental factors. Moreover, incorporating such samples could validate the observed molecular and functional insights, bridging the gap between experimental findings and their potential translational impact.

## Conclusion

In conclusion, our findings underscore the importance of *MIR31HG* as a key regulator of PDAC progression, particularly in the basal-like subtype, and suggest that targeting *MIR31HG* could be a promising approach to mitigate PDAC aggressiveness and improve patient outcomes. Further research is needed to fully elucidate the molecular mechanisms of *MIR31HG* in PDAC and to develop targeted therapies that can effectively disrupt its oncogenic functions.

## Electronic supplementary material

Below is the link to the electronic supplementary material.

Supplementary Material 1

## Data Availability

The datasets used and analyzed during the current study are available from the corresponding author on reasonable request.

## Abbreviations

BNIP3: BCL2 interacting protein 3
CA9: carbonic anhydrase 9
CDH1: E-cadherin
COL1A1: collagen type I alpha 1 chain
ECM: extracellular matrix
EMT: Epithelial-mesenchymal transition
FOLFIRINOX: a combination of fluorouracil, leucovorin, irinotecan, and oxaliplatin
GATA6: GATA binding protein 6
GSEA: gene set enrichment analysis
HNF1A: HNF1 homeobox A
KRT5: keratin 5
LDHA: lactate dehydrogenase A
LOXL2: lysyl oxidase like 2
MIR31HG: MIR31 host gene
MYC: MYC proto-oncogene, bHLH transcription factor
PAAD: pancreatic adenocarcinoma
PDAC: pancreatic ductal adenocarcinoma, qPCR, real-time quantitative polymerase chain reaction
TCGA: The Cancer Genome Atlas
VIM: vimentin
