# Supplementary material for: Hypoxia-induced MIR31HG expression promotes partial EMT and basal-like phenotype in pancreatic ductal adenocarcinoma based on data mining and experimental analyses
Source: J Transl Med. 2025 Mar 10;23:305. doi: 10.1186/s12967-025-06292-x (PMC11895263; doi:10.1186/s12967-025-06292-x)
Supplement: Supplementary file 1 — Supplementary Material 1 [file 12967_2025_6292_MOESM1_ESM.pdf]

Supplementary Data

Hypoxia-induced MIR31HG expression promotes partial EMT and basal-like phenotype in pancreatic ductal adenocarcinoma based on data mining and experimental analyses

Ching-Chung Ko [1,2,3] and Pei-Ming Yang [4,5,6,7,8,*]

[1]Department of Medical Imaging, Chi Mei Medical Center, Tainan 71004, Taiwan
[2]Department of Health and Nutrition, Chia Nan University of Pharmacy and Science, Tainan 71710, Taiwan
[3]School of Medicine, College of Medicine, National Sun Yat-Sen University, Kaohsiung 80424, Taiwan
[4]Graduate Institute of Cancer Biology and Drug Discovery, College of Medical Science and Technology, Taipei Medical University, Taipei 11031, Taiwan
[5]PhD Program for Cancer Molecular Biology and Drug Discovery, College of Medical Science and Technology, Taipei Medical University and Academia Sinica, Taipei 11031, Taiwan
[6]TMU Research Center of Cancer Translational Medicine, Taipei 11031, Taiwan
[7]Cancer Center, Wan Fang Hospital, Taipei Medical University, Taipei 11696, Taiwan
[8]Taipei Medical University (TMU) and Affiliated Hospitals Pancreatic Cancer Groups, Taipei Cancer Center, Taipei Medical University, Taipei 11031, Taiwan

***To whom correspondence should be addressed:**
Pei-Ming Yang, PhD

Graduate Institute of Cancer Biology and Drug Discovery, College of Medical Science and Technology, Taipei Medical University, No. 301, Yuantong Rd., Zhonghe Dist., New Taipei City 235603, Taiwan; Phone: 886-2-66202589 ext. 11107; E-mail: yangpm@tmu.edu.tw

Supplementary Data include:
Supplementary Materials and Methods
Supplementary Figures S1-S3

Supplementary Materials and Methods

Western blot analysis

Total protein lysates were extracted using the RIPA lysis and extraction buffer (#89901; Thermo Fisher Scientific, Waltham, MA, USA) supplemented with a 1× concentration of protease (#11873580001) and phosphatase (#04906837001) inhibitor cocktails (Roche, Indianapolis, IN, USA). Proteins were then separated via sodium dodecyl sulfate-polyacrylamide gel electrophoresis (SDS-PAGE) and subsequently transferred onto nitrocellulose membranes. The membranes were blocked with 5% skimmed milk in TBST buffer (20 mM Tris-base, 150 mM NaCl, and 0.05% Tween-20). They were then incubated with a specific primary antibody followed by a horseradish peroxidase (HRP)-conjugated secondary antibody. Protein bands were visualized using the Western Lightning Plus ECL detection reagent (#NEL105001EA; PerkinElmer, Waltham, MA, USA) and detected with the GE Amersham Imager 600 (GE Healthcare Life Sciences, Marlborough, MA, USA). E-cadherin antibody (#3195) was purchased from Cell Signaling Technology (Berkeley, CA, USA). Vimentin (#GTX100619), LAMB3 (#GTX103736), and LAMC2 (#GTX113765) antibodies were purchased from GeneTex (Hsinchu City, Taiwan). HRP-conjugated anti-rabbit secondary antibody (#111-035-003) was from Jackson ImmunoResearch (West Grove, PA, USA).

Supplementary Figures

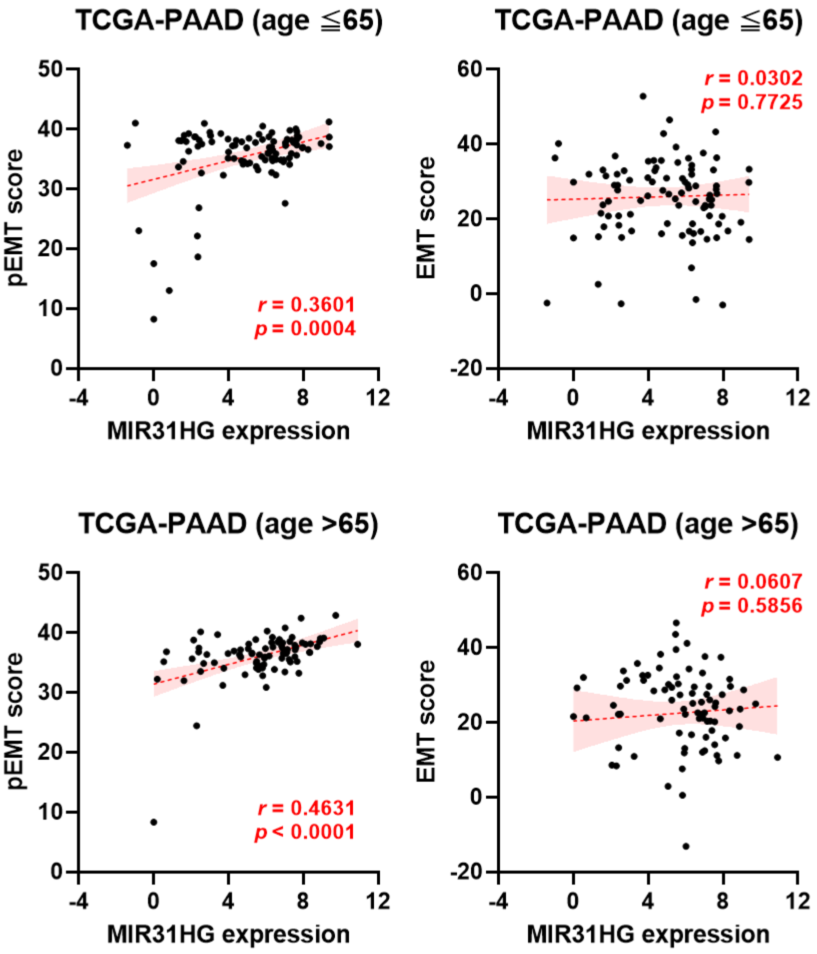

Supplementary Figure S1. The correlation between MIR31HG and partial EMT in PDAC patients of different age groups. Data from PDAC patients (TCGA-PAAD dataset; n = 177) were obtained from the cBioPortal website (https://www.cbioportal.org/). Patients were divided into two age groups: age ≦65 (n = 94) and >65 (n = 83). EMT and partial EMT (pEMT) scores were calculated as described in the Materials and Methods section, and their correlation with MIR31HG gene expression was analyzed.

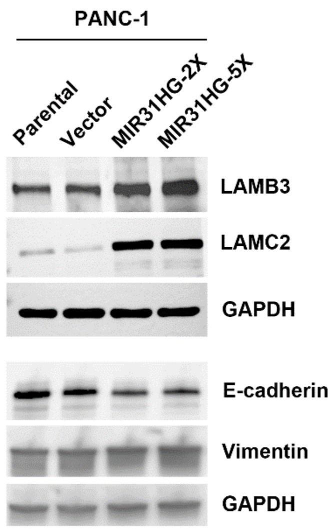

Supplementary Figure S2. Effect of MIR31HG overexpression on EMT and partial EMT marker expression. The protein expression levels of EMT markers (E-cadherin and vimentin) and partial EMT markers (LAMB3 and LAMC2) in parental PANC-1, PANC-1-Vector, PANC-1-MIR31HG-2X, and PANC-1-MIR31HG-5X cells were analyzed by western blotting.

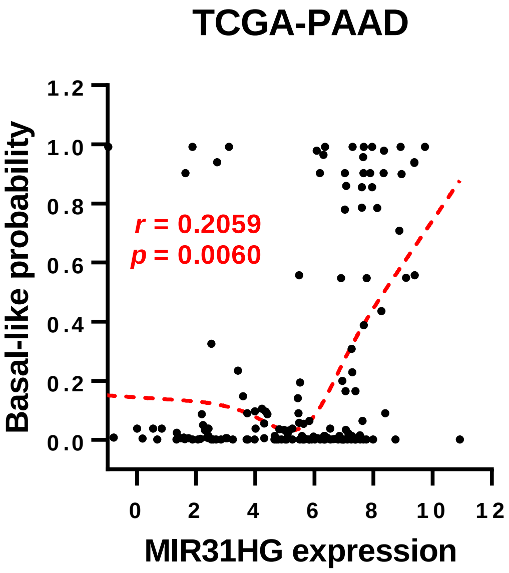

Supplementary Figure S3. The correlation between MIR31HG and basal-like probability in PDAC patients. (A) Data from PDAC patients (TCGA-PAAD dataset; n = 177) were obtained from the cBioPortal website (https://www.cbioportal.org/). The basal-like probability was calculated using the PurIST classifier and plotted against MIR31HG expression. Their relationship was analyzed using Spearman's rank correlation.
